# Effectiveness of nirmatrelvir/ritonavir and molnupiravir on post-COVID diabetes risk among an older adult cohort: a target trial emulation study

**DOI:** 10.1186/s12916-026-04791-2

**Published:** 2026-03-17

**Authors:** Zihao Guo, Yuchen Wei, Aimin Yang, Carlos King Ho Wong, Xi Xiong, Kailu Wang, Guozhang Lin, Huwen Wang, Chi Tim Hung, Conglu Li, Carrie Ho Kwan Yam, Tsz Yu Chow, Shi Zhao, Chris Ka Pun Mok, David S. C. Hui, Eng Kiong Yeoh, Ka Chun Chong

**Affiliations:** 1 The Jockey Club School of Public Health and Primary Care, The Chinese University of Hong Kong, Hong Kong, China; 2 Department of Medicine & Therapeutics, Faculty of Medicine, The Chinese University of Hong Kong, Hong Kong Special Administrative Region, China; 3 Laboratory of Data Discovery for Health, Hong Kong Science Park, Hong Kong Special Administrative Region, China; 4 Department of Family Medicine and Primary Care, School of Clinical Medicine, Faculty of Medicine, Li Ka Shing, The University of Hong Kong, Hong Kong Special Administrative Region, China; 5 Department of Infectious Disease Epidemiology, School of Hygiene and Tropical Medicine, London, UK; 6 Research Department of Practice and Policy, School of Pharmacy, University College London, London, UK; 7 Duke-NUS Medical School, Singapore, Singapore; 8 School of Public Health, Tianjin Medical University, Tianjin, China; 9 Li Ka Shing Institute of Health Sciences, Chinese University of Hong Kong, Hong Kong, China; 10 School of Biomedical Sciences, The Chinese University of Hong Kong, Hong Kong SAR, China; 11 S.H. Ho Research Centre for Infectious Diseases, Chinese University of Hong Kong, Hong Kong, China

**Keywords:** Diabetes, Hong Kong, Molnupiravir, Nirmatrelvir/ritonavir, Post-acute COVID sequelae, SARS-CoV-2, Target trial emulation

## Abstract

**Background:**

Accumulating evidence indicates that SARS-CoV-2 infection is associated with a broad spectrum of post-acute COVID sequelae, including diabetes. While nirmatrelvir/ritonavir and molnupiravir have demonstrated efficacy in reducing acute COVID-19 severity, their protective effects against post-COVID diabetes remain uncertain. In this study, we aimed to evaluate the effectiveness of these antiviral agents in reducing post-COVID diabetes risks, including new-onset diabetes in non-diabetic individuals and exacerbated diabetes in those with pre-existing diabetes.

**Methods:**

We emulate target randomized controlled trials of COVID-19 antivirals in hospitalized patients who tested positive for SARS-CoV-2 between March 11, 2022, and October 10, 2023, in Hong Kong. Two analytic patient cohorts for assessing incident diabetes and exacerbation of diabetes for rehospitalization, including those with or without diabetes confirmed before the index date, were identified. Cloning, censoring, and weighting were used to emulate the target trials of nirmatrelvir/ritonavir and molnupiravir, involving treatment arm and control arm within each trial. Cause-specific Cox proportional hazard model and an extended form of Cox model for modeling recurrent hospitalizations were used to estimate the hazard ratio (HR) between arms in each trial, adjusting for baseline covariates.

**Results:**

Among 88,643 hospitalized patients first time infected by SARS-CoV-2 identified, 35,997 and 18,865 eligible patients were included in the two analytic cohorts for the analysis on newly onset diabetes and exacerbated diabetes for rehospitalization, respectively. The median follow-up period ranged from 344 to 365 days across treatment and control arms of target trials. Compared with the no treatment arm, non-diabetic patients who received nirmatrelvir/ritonavir showed a significantly lower risk of post-COVID incident diabetes (HR: 0.75, 95% CI: 0.61 to 0.92). A reduced risk of diabetes rehospitalizations (HR: 0.70, 95% CI: 0.60 to 0.81) was observed among the diabetic patients. No significant associations were found for the use of molnupiravir and post-COVID diabetes outcomes.

**Conclusions:**

Our study demonstrates the effectiveness of nirmatrelvir/ritonavir in reducing the risks of post-acute COVID sequelae of diabetes in the hospitalized population, regardless of their diabetic status, whereas molnupiravir showed no significant benefit. Our findings offer valuable clinical insights for managing diabetes during the post-acute phase of SARS-CoV-2 infection.

**Supplementary Information:**

The online version contains supplementary material available at 10.1186/s12916-026-04791-2.

## Background

SARS-CoV-2 infection results in not just acute illness but also long-term sequelae. It may initiate or worsen post-COVID chronic conditions, including neurological, cardiovascular, and cardiometabolic diseases [1–3]. According to the systematic reviews [4, 5], COVID-19 was associated with a higher risk of newly diagnosed diabetes, compared to individuals without COVID-19. The findings were generally consistent among different populations, such as children [6]. In addition, Xie and Al-Aly [3] demonstrated an increased risk of incident diabetes in the post-acute phase of COVID-19 among the survivors after 30 days of SARS-CoV-2 infection.

Nirmatrelvir/ritonavir and molnupiravir have been approved for the antiviral treatment of COVID-19 since 2021. Studies have consistently demonstrated the short-term effectiveness of nirmatrelvir/ritonavir and molnupiravir among high-risk outpatients [7–9]. In addition, investigations have shown long-term benefits of the antivirals on post-COVID sequelae in various populations [10–14], although small trials were unable to demonstrate a significant benefit in improving long COVID symptoms [15, 16]. Studies have shown that the use of nirmatrelvir/ritonavir was not associated with a significantly lower risk of developing diabetes within 6 months post-infection among non-hospitalized patients with COVID-19 who were free from diabetes [17, 18]. In COVID-19 patients with diabetes, several investigations have examined the short-term effectiveness of antivirals [19–22]. Nevertheless, not only is there a lack of evaluation on the long-term effects, but none has assessed the antiviral effectiveness against newly onset diabetes in COVID-19 hospitalizations without diabetes, nor exacerbated diabetes in those with pre-existing diabetes.

Since post-COVID diabetes is more common in patients with severe SARS-CoV-2 infections [23] (e.g., those requiring hospitalizations) compared to mild infections, we hypothesize that antivirals prescribed during the acute phase may lower the risk of post-acute incident diabetes in hospitalized patients without diabetes and reduce exacerbations in those with diabetes, given the mitigating effects of the antivirals on acute severity. In this study, we used a target trial emulation study to examine the effectiveness of nirmatrelvir/ritonavir and molnupiravir on the post-COVID diabetes risk, including new-onset diabetes in non-diabetic individuals and exacerbated diabetes in those with pre-existing diabetes.

## Methods

### Study design

We employed a population-based, retrospective cohort of hospitalized patients with positive SARS-CoV-2 reverse transcription polymerase chain reaction (RT-PCR) test results to emulate target trials of first-line COVID-19 antivirals—nirmatrelvir/ritonavir and molnupiravir. The direct use of observational data in a cohort study design often fails to align the time of meeting the eligibility criteria with the assignment of treatment strategies, which may introduce selection bias and immortal-time bias [24]. Applying a target trial emulation design to real-world data could circumvent these biases by using a cloning-censoring-weighting procedure to mimic clinical trial conditions, while preserving the advantages of large observational databases and strengthening the comparability of treatment and control groups [25]. Four target trials were emulated for the outcomes of post-COVID diabetes, including newly onset diabetes and exacerbated diabetes for rehospitalizations. The specification of the target trials was detailed in Additional file 1: Table S1.

### Study setting and data sources

Anonymized electronic health records (EHR) collected from the Hospital Authority (HA) and the Department of Health (DH) in Hong Kong were used in this study. As a statutory body, the HA delivers public inpatient and outpatient care, serving over 7.3 million residents and handling more than 90% of all hospitalizations in the region. Additionally, more than 90% of patients with diabetes were managed by the public health systems of HA. Patients’ EHR, including demographic information, death registry data, inpatient and outpatient records, laboratory test results, and medication prescriptions, were obtained from the HA database. DH provided de-identified population-based vaccination records, which were matched to the individual EHR with unique pseudo codes.

The study followed the TARGET (The Transparent Reporting of Observational Studies Emulating a Target Trial) guideline [26] and the STROBE (Strengthening the Reporting of Observational Studies in Epidemiology) reporting guideline. Ethics approval was obtained from the Joint CUHK-NTEC Clinical Research Ethics Committee (No. 2023.006). No study protocol was registered for this study.

### Study participants

We constructed two analytic cohorts of patients aged 18 years or older who were first-time infected with SARS-CoV-2 confirmed by positive reverse transcription polymerase chain reaction (RT-PCR) results obtained from March 11, 2022, to October 10, 2023 (21 days before the end of the data availability date, which is October 31, 2023). During the enrolment period, the SARS-CoV-2 Omicron variants were the dominant circulating variants in Hong Kong. Patients who were admitted within 3 days before or after the initial positive RT-PCR date were considered as COVID-19 hospitalizations and were eligible for inclusion [12, 13]. This inclusion criteria also took into account the possible delay between case confirmation and hospital admission during the growth phase of the epidemic. The index date was defined as the earliest calendar date when the subject was RT-PCR test-positive for SARS-CoV-2 infection. The indication of nirmatrelvir/ritonavir and molnupiravir was initiation within 5 days of the symptom onset (the index date was used as a surrogate of the symptom onset date). For nirmatrelvir/ritonavir target trials, patients with the following contraindications to nirmatrelvir/ritonavir were additionally excluded: (1) drug contraindications (e.g., amiodarone, lumacaftor–ivacaftor, rifampicin, apalutamide, phenobarbital, rifapentine, carbamazepine, phenytoin, St John’s wort [*Hypericum perforatum*], ivosidenib, and primidone) within 90 days of the index date; (2) severe renal impairment (i.e., estimated glomerular filtration rate < 30 mL/min per 1.73 m^2^, dialysis, or renal transplantation); (3) severe liver impairment (i.e., cirrhosis, hepatocellular carcinoma, or liver transplantation) [8, 27].

The first analytic cohort was formed to assess the effectiveness of antiviral drugs against incident post-acute COVID-19 diabetes, specifically among patients without a prior diagnosis of diabetes before the index date. The second analytic cohort, which evaluated antiviral effectiveness against exacerbated diabetes leading to rehospitalization, consisted of patients with a pre-existing diabetes diagnosis before the index date. Patients with diabetes was identified based on International Classification of Diseases, Ninth Revision, Clinical Modification (ICD-9-CM) code (250.xx), the International Classification of Primary Care, Second Edition (ICPC-2) code (T89 or T90), an HbA_1c_ measurement of ≥ 6.5%, or prescription records of anti-diabetic medication, in line with previous studies [3, 28–31]. Patients with a diagnosis of type 1 diabetes, who constituted less than 0.5% of the second analytic cohort, were excluded to facilitate a clearer interpretation of the results related to type 2 diabetes.

To assess the robustness of the definition of exacerbated diabetes in the second analytic cohort, we also conducted a post hoc analysis using a composite dichotomous measure of diabetes progression [32]. This outcome definition consisted of (1) therapy intensification, defined as either the initiation of insulin or an increase in the number of glucose-lowering medication classes used during follow-up compared to the number used in the year before the index date, and (2) a new diagnosis of acute glycemic complications during follow-up, including diabetic ketoacidosis or uncontrolled diabetes, in patients with no record of such complications in the year before the index date.

### Outcomes

The outcome of the first analytic cohort was post-COVID newly onset diabetes and the outcome of the second analytic cohort was exacerbated diabetes rehospitalizations, identified based on the diagnosed code at admission (ICD-9-CM). Incident post-acute COVID-19 diabetes was assessed in the period of follow-up from 21 days after the index date up to the occurrence of outcome events, death, 365 days after the index date, or the end of data availability, whichever came first. The 21-day threshold was selected as it is a commonly adopted definition of the post-acute phase of SARS-CoV-2 infection in previous studies conducted in the Hong Kong population [12, 13, 33, 34], which facilitates comparisons of our results with those studies. For identifying exacerbated diabetes for rehospitalization, the follow-up was not censored upon the occurrence of the outcome, thereby enabling evaluation of recurrent event rates over time while retaining the same end-of-follow-up criteria as in the analysis for the first analytic cohort.

### Covariates

We collected baseline characteristics of eligible patients, including age; sex; Charlson Comorbidity Index (CCI, derived from prior diagnoses); pre-existing conditions (congestive heart failure, cerebrovascular disease, peripheral vascular disease, chronic pulmonary disease, dementia, cancer, liver disease, renal disease, hypertension, peptic ulcer disease, and rheumatic disease); immunocompromised status; intensive care unit admission and ventilation support (Additional file 1: Table S2) within 3 days post index date; concomitant treatments initiated within 3 days after index date (dexamethasone, methylprednisolone, prednisolone, interferon-beta-1b, baricitinib, tocilizumab, remdesivir); COVID-19 vaccination status (unvaccinated, 1–2 doses, ≥ 3 doses); indicators of healthcare service utilizations in pre-pandemic phase, including any general outpatient visit and hospital admission during 2018–2019; use of lipid-lowering drugs and angiotensin-converting enzyme inhibitors (ACEI) within 1 year before index date [28, 35]; and calendar week of the index date. For analyses of diabetes exacerbation leading to rehospitalization, years of diabetes diagnosis before the index date was also included as a covariate.

Vaccinated patients were defined as those who received the COVID-19 vaccine at least 14 days before the index date. The immunocompromised patients were those with diagnosed immunocompromising conditions (HIV, hematological malignancy, immune-mediated rheumatic disease, other hematological conditions, solid organ transplant, and bone marrow or stem cell transplant). Patients were also considered as immunocompromised if they had a history of receiving or had remaining days supply of a monoclonal antibody within the last 3 months, an oral immunosuppressive drug within the last month, an oral glucocorticoid (equivalent to 20 mg/day of prednisone taken continuously) within the last month, or if they had received an immunosuppressive infusion or injection within the 3 months before the index date [13].

### Statistical analysis

We implemented the target trial emulation through a cloning, censoring, and weighting framework following previous works [14, 25]. Specifically, for each trial, all eligible patients were first cloned at a 1:1 ratio to either the treatment arm or the control arm. Censoring was then performed for clones if they deviated from the treatment protocol during a 5-day grace period following SARS-CoV-2 diagnosis (index date). This grace period aligns with antiviral treatment guidelines from the DH of Hong Kong and the US Food and Drug Administration, which recommend initiating nirmatrelvir/ritonavir or molnupiravir within 5 days of illness onset [36, 37]. Therefore, the treatment strategies compared in this study were (1) receiving nirmatrelvir/ritonavir or molnupiravir within 5 days of the index date (treatment) and (2) not receiving either antiviral within the 5-day grace period (control), which included patients who never initiated treatment or initiated it after 5 days. At each day of the grace period, clones in any of the antiviral treatment arms were censored if they did not receive the corresponding treatment, while clones in the control arm were censored when they received either of the treatments. This procedure ensures that all study participants follow the assigned treatment strategy within the 5-day grace period post index date, thereby addressing the immortal-time bias. An inverse probability of censoring weighting procedure (IPCW) was performed at each day to deal with the selection bias resulting from the censoring, where the probability of not being censored at each day was calculated by a multivariate logistic regression model adjusting for baseline covariates, conditional on the remaining uncensored on the previous day. The inverse of the probability was further stabilized by the proportion of individuals remaining in the group, which was considered as the weight on that day. The cumulative multiplicative weights on the last day of the 5-day grace period were used for further analyses. Covariates with an absolute standardized mean difference (SMD) < 0.1 after IPCW were deemed as well-balanced between arms [38].

To compare observed post-acute COVID diabetes outcomes between the treatment arm and the control arm, the crude cumulative incidence and incidence rate difference (IRD) per 1000 person-years were calculated before IPCW. Given death as a competing event, the cause-specific Cox proportional hazard model was used to estimate the adjusted hazard ratio (HR) between arms in each trial after IPCW [39, 40]. For the analysis on exacerbated diabetes for rehospitalizations, we used an Andersen-Gill counting process model—a generalized form of the Cox model—to account for dependence between repeated hospitalizations within individuals [41] (Additional file 1: Fig. S1). In addition, the sandwich estimator was adopted to generate robust standard errors, ensuring accurate variance and CI estimation in the model, considering potential independence between individuals caused by IPCW. The proportional hazards assumption was assessed by visually inspecting plots of scaled Schoenfeld residuals against transformed time.

Subgroup analyses were conducted based on age groups (≤ 65 years or > 65 years), sex (female or male), vaccination status (unvaccinated or ≥ 1 dose of vaccine), and CCI categories (< 4 or ≥ 4). We conducted interaction analysis by computing the relative excess risk for interaction (RERI) to evaluate the additive interaction [13], and the exponential of the coefficient of the product term as the measurement of multiplicative interaction.

To examine the robustness of the primary results, we conducted several sensitivity analyses by (1) excluding patients who initiated nirmatrelvir/ritonavir or molnupiravir beyond 5 days of the index date; (2) excluding patients who initiated either antiviral treatment after 3 days of the index date; (3) excluding patients who were admitted to hospital before initial positive RT-PCR test, which was considered as a proxy of admission related to COVID-19 symptomatology; (4) excluding patients who received remdesivir; (5) truncating IPCW at the 1 st and 99th percentiles [42]; and (6) applying the Prentice, Williams, and Peterson repeated events model for total time (PWP-TT) as an alternative method for modeling the recurrent diabetes rehospitalizations [41].

Furthermore, we employed a negative control of outcome approach to assess the extent to which the treatment and control arms were comparable regarding the propensity for developing outcomes that were implausible to be associated with treatment strategy and SARS-CoV-2 infection [43]. Negative control of outcome included injury, trauma, or poisoning (ICD-9 CM: 800–999 and E000–E999; ICPC-2: A80, D80, F79, H78, H79, L81, N80, N81, R88, S19, U80, W75, Y80, and X82). To mitigate the influence of prior events, we excluded patients with these diagnoses within 1 year before the index date. This approach ensured that covariate sets remained consistent with those used for the primary outcome (post-COVID diabetes) [43].

For all analysis, non-parametric bootstrap with 1000 resamples was applied to compute 95% confidence intervals (CIs). All statistical tests were two-tailed, and a *p* value < 0.05 was considered as a sign of statistical significance.

All statistical analyses were performed by using R statistical software (version 4.4.2) (R Program for Statistical Computing).

## Results

**Fig. 1:**
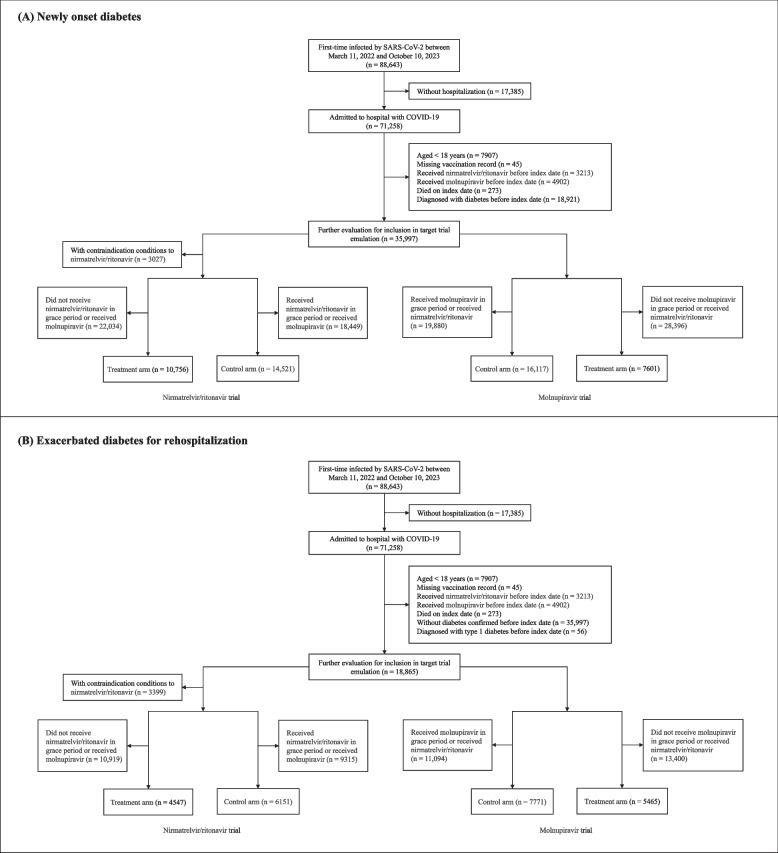
Flowchart of patients’ inclusion and exclusion for two analytic cohorts

A total of 88,643 patients first time infected by SARS-CoV-2 and were admitted to hospital were identified from March 11, 2022, to October 10, 2023. Among them, 35,997 and 18,865 patients were eligible for inclusion in the two analytic cohorts for the analysis on newly onset diabetes and exacerbated diabetes for rehospitalization. After the cloning-censoring-weighting procedure, 25,277 patients were included in the nirmatrelvir/ritonavir trial, and 23,718 were included in the molnupiravir trial at the end of the grace period, respectively; for the second analytic cohort, 10,698 patients were included in the nirmatrelvir/ritonavir trial and 13,236 were included in the molnupiravir trial at the end of the grace period, respectively (Fig. 1).

The baseline characteristics of patients across the target trials of the two analytic cohorts were shown in Table 1. In analytic cohort for assessing newly onset diabetes, patients in treatment arm and control arm of nirmatrelvir/ritonavir trial had a mean age of 73 (SD: 16.0) years and 67.5 (22.2) years, with 5160 (48.0%) and 7616 (52.4%) were female patients, respectively; patients in molnupiravir trial had a mean age of 76.3 (16.4) years and 68.7 (22.0) years, with 3741 (49.2%) and 8367 (51.9%) were female patients, respectively; in analytic cohort for analyzing exacerbated diabetes for rehospitalization, patients in nirmatrelvir/ritonavir trial had a mean age of 76.7 (11.1) years and 77.3 (12.8) years, with 2177 (47.9%) and 2738 (44.5%) were female patients, respectively; patients in molnupiravir trial had a mean age of 77.9 (12.0) years and 77.6 (12.7) years, with 2587 (47.3%) and 3490 (44.9%) female patients, respectively. After IPCW, all baseline covariates were well balanced between arms in each trial, with an SMD of less than 0.1 (Additional file 1: Figs. S2–S5). The proportional hazards assumption was tested and showed no clear evidence of violations (Additional file 1: Fig. S6). All of the incident diabetes outcomes were identified as type 2 diabetes based on the ICD-9 CM and ICPC-2 code.

**Table 1:** Baseline characteristics of participants included in target trials before IPCW

Characteristics	Newly onset diabetes						Exacerbated diabetes for rehospitalization					
	Nirmatrelvir/ritonavir trial		SMD^†^	Molnupiravir trial		SMD^†^	Nirmatrelvir/ritonavir trial		SMD^†^	Molnupiravir trial		SMD^†^
	Control (*n* = 14,521)	Treatment (*n* = 10,756)		Control (*n* = 16,117)	Treatment (*n* = 7601)		Control (*n* = 6151)	Treatment (*n* = 4547)		Control (*n* = 7771)	Treatment (*n* = 5465)	
Age	67.5 (22.2)	73.0 (16.0)	0.00	68.7 (22.0)	76.3 (16.4)	0.03	77.3 (12.8)	76.7 (11.1)	− 0.02	77.6 (12.7)	77.9 (12.0)	0.01
Sex			0.01			0.02			0.00			0.00
Female	7616 (52.4)	5160 (48.0)		8367 (51.9)	3741 (49.2)		2738 (44.5)	2177 (47.9)		3490 (44.9)	2587 (47.3)	
Male	6905 (47.6)	5596 (52.0)		7750 (48.1)	3860 (50.8)		3413 (55.5)	2370 (52.1)		4281 (55.1)	2878 (52.7)	
Vaccination status												
Unvaccinated	4353 (30.0)	1667 (15.5)	− 0.01	5074 (31.5)	1688 (22.2)	0.03	2080 (33.8)	646 (14.2)	− 0.02	2773 (35.7)	1247 (22.8)	0.02
1–2 doses	4527 (31.2)	2025 (18.8)	0.00	5030 (31.2)	1716 (22.6)	0.00	2171 (35.3)	879 (19.3)	− 0.01	2696 (34.7)	1283 (23.5)	0.00
≥ 3 doses	5641 (38.8)	7064 (65.7)	0.01	6013 (37.3)	4197 (55.2)	− 0.02	1900 (30.9)	3022 (66.5)	0.02	2302 (29.6)	2935 (53.7)	− 0.01
Charlson Comorbidity Index	1 (1-1)	1 (1-1)	0.01	1 (1-1)	1 (1, 2)	0.02	1 [0–1]	1 [0–1]	0.00	1 [0–1]	1 [0–2]	0.03
Pre-existing conditions												
Myocardial infarction	94 (0.6)	51 (0.5)	0.00	108 (0.7)	91 (1.2)	0.00	78 (1.3)	35 (0.8)	0.00	113 (1.5)	121 (2.2)	0.00
Congestive heart failure	212 (1.5)	97 (0.9)	0.00	254 (1.6)	184 (2.4)	0.00	194 (3.2)	69 (1.5)	0.00	267 (3.4)	227 (4.2)	0.00
Peripheral vascular disease	128 (0.9)	76 (0.7)	0.00	158 (1.0)	82 (1.1)	0.00	109 (1.8)	42 (0.9)	0.00	150 (1.9)	90 (1.6)	0.00
Cerebrovascular disease	1193 (8.2)	786 (7.3)	0.00	1410 (8.7)	968 (12.7)	0.00	862 (14.0)	544 (11.9)	0.00	1117 (14.4)	862 (15.8)	0.01
Chronic pulmonary disease	1109 (7.6)	537 (5.0)	0.00	1222 (7.6)	467 (6.1)	0.00	453 (7.4)	186 (4.1)	0.00	553 (7.1)	304 (5.6)	0.00
Hypertension	4596 (31.7)	4122 (38.3)	0.00	5290 (32.8)	3239 (42.6)	0.01	3965 (64.5)	3076 (67.6)	0.00	4964 (63.9)	3510 (64.2)	0.00
Liver disease	330 (2.3)	178 (1.7)	0.00	451 (2.8)	287 (3.8)	0.00	216 (3.5)	112 (2.5)	0.00	369 (4.7)	297 (5.4)	0.00
Peptic ulcer disease	41 (0.3)	29 (0.3)	0.00	46 (0.3)	19 (0.2)	0.00	17 (0.3)	15 (0.3)	0.00	19 (0.2)	12 (0.2)	0.00
Rheumatic disease	119 (0.8)	93 (0.9)	0.00	139 (0.9)	106 (1.4)	0.00	55 (0.9)	26 (0.6)	0.00	65 (0.8)	35 (0.6)	0.00
Renal disease	115 (0.8)	53 (0.5)	0.00	180 (1.1)	184 (2.4)	0.00	166 (2.7)	67 (1.5)	− 0.01	292 (3.8)	336 (6.1)	0.00
Dementia	222 (1.5)	113 (1.1)	0.00	250 (1.6)	174 (2.3)	0.00	120 (1.9)	60 (1.3)	− 0.01	143 (1.8)	104 (1.9)	0.01
Cancer	211 (1.5)	158 (1.5)	0.00	231 (1.4)	110 (1.4)	0.00	104 (1.7)	78 (1.7)	0.00	133 (1.7)	82 (1.5)	0.00
Immunocompromised status	826 (5.7)	630 (5.9)	0.00	940 (5.8)	516 (6.8)	0.01	372 (6.0)	202 (4.4)	0.00	478 (6.2)	314 (5.7)	0.00
Concomitant pharmacological treatments												
Dexamethasone	4821 (33.2)	1257 (11.7)	0.03	5650 (35.1)	1675 (22.0)	0.05	2815 (45.8)	611 (13.4)	0.01	3590 (46.2)	1172 (21.4)	0.04
Methylprednisolone	26 (0.2)	7 (0.1)	0.00	28 (0.2)	4 (0.1)	0.00	9 (0.1)	2 (< 0.1)	0.00	16 (0.2)	2 (< 0.1)	0.00
Prednisolone	870 (6.0)	448 (4.2)	0.00	968 (6.0)	428 (5.6)	0.00	389 (6.3)	153 (3.4)	0.00	483 (6.2)	308 (5.6)	0.00
Baricitinib	336 (2.3)	123 (1.1)	0.01	402 (2.5)	88 (1.2)	0.00	218 (3.5)	58 (1.3)	0.01	294 (3.8)	67 (1.2)	0.00
Tocilizumab	162 (1.1)	37 (0.3)	0.00	191 (1.2)	41 (0.5)	0.00	103 (1.7)	17 (0.4)	0.00	138 (1.8)	27 (0.5)	0.00
Remdesivir	3284 (22.6)	817 (7.6)	0.04	3706 (23.0)	975 (12.8)	0.05	1969 (31.9)	390 (8.6)	0.04	2407 (31.0)	722 (13.2)	0.05
Interferon	97 (0.7)	7 (0.1)	0.00	120 (0.7)	36 (0.5)	0.00	55 (0.9)	3 (0.1)	0.00	72 (0.9)	12 (0.2)	0.00
Concomitant non-pharmacological treatments												
Intensive care unit admission	399 (2.7)	100 (0.9)	0.01	473 (2.9)	104 (1.4)	0.00	332 (5.4)	74 (1.6)	0.01	477 (6.1)	81 (1.5)	0.01
Use of ventilatory support	481 (3.3)	90 (0.8)	0.00	602 (3.7)	109 (1.4)	0.01	311 (5.0)	48 (1.1)	0.00	457 (5.9)	102 (1.9)	0.01
Healthcare utilization in pre-pandemic phase												
Any GOPC attendance	277 (1.9)	294 (2.7)	0.00	299 (1.9)	136 (1.8)	0.00	196 (3.2)	198 (4.4)	0.00	231 (3.0)	120 (2.2)	0.00
Any hospital admission	353 (2.4)	95 (0.9)	0.00	419 (2.6)	92 (1.2)	0.00	158 (2.6)	40 (0.9)	0.00	214 (2.8)	100 (1.8)	0.00
Prior use of lipid-lowering drugs	4009 (27.6)	3372 (31.3)	0.00	4627 (28.7)	3561 (46.8)	0.00	4510 (73.1)	3470 (76.0)	0.01	5680 (73.1)	4294 (78.6)	0.03
Years since diagnosis of diabetes	–	–	–	–	–	–	3.1 (1.4)	3.5 (1.4)	0.02	3.1 (1.4)	3.3 (1.3)	0.00
Prior use of ACEI	1535 (10.6)	1028 (9.6)	0.00	1798 (11.2)	1207 (15.9)	0.00	1833 (29.8)	1393 (30.6)	− 0.01	2328 (30.0)	1748 (32.0)	0.00

*IPCW*, inverse probability of censoring weighting; *SMD*, standardized mean difference; *GOPC*, general outpatient clinics; *ACEI*, angiotensin-converting enzyme inhibitors

Data are presented in *n* (%), median [IQR], or mean (SD)

^†^Standardized mean difference after IPCW

### Effect of nirmatrelvir/ritonavir

**Fig. 2:**
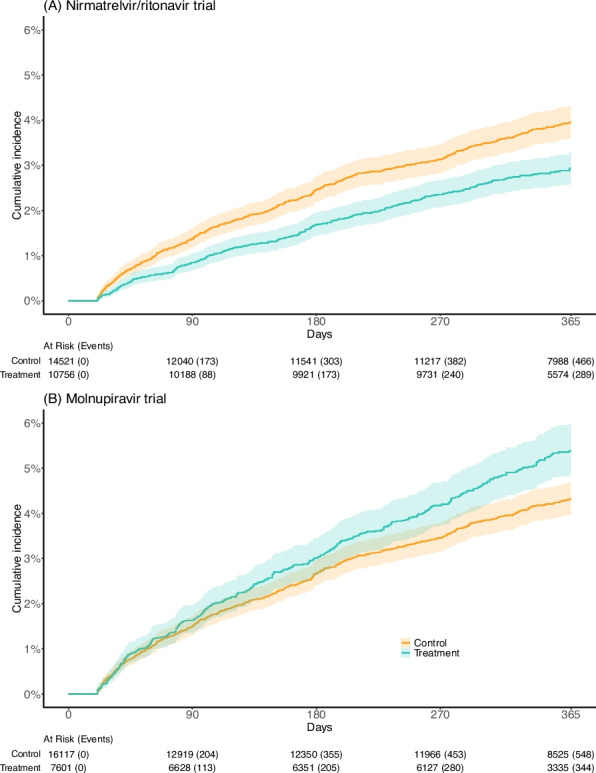
Unadjusted cumulative incidence of newly onset diabetes between treatment arm (green solid lines) and control arm (yellow solid lines) of the emulated target trials. Shaded regions indicated 95% confidence bands. Tables represented the number of patients at risk and events observed during the follow-up period

**Fig. 3:**
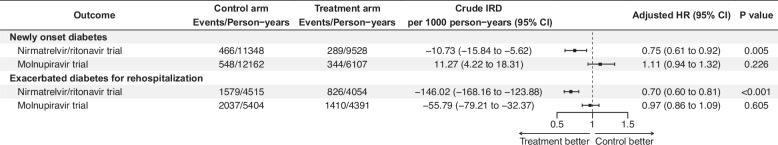
Risk of post-acute COVID diabetes outcomes in emulated target trials. Crude incidence rate difference was calculated for treatment arm and control arm before inverse probability of censoring weighting. Adjusted hazard ratios were estimated using weighted cause-specific Cox proportional hazard models. IRD, incidence rate difference; HR, hazard ratio; CI, confidence interval

For the analysis on newly onset diabetes, 10,756 were in the treatment arm and 14,521 were in the control arm of the nirmatrelvir/ritonavir trial; the median follow-up time was 365 days [IQR: 317–365] and 365 days [291–365], corresponding to 9528 person-years and 11,348 person-years of follow-up, respectively. The crude cumulative incidence of newly onset diabetes in the nirmatrelvir/ritonavir trial was shown in Fig. 2A. The crude IRD between two arms for newly onset diabetes was − 10.73 (− 15.84 to − 5.62) per 1000 person-years. Compared with no treatment, the use of nirmatrelvir/ritonavir was associated with a significantly lower risk of post-COVID incident diabetes (HR = 0.75; 95% CI: 0.61 to 0.92, *p* = 0.005) (Fig. 3).

Analyses on exacerbated diabetes for rehospitalization included 4547 patients in the nirmatrelvir/ritonavir treatment arm and 6151 patients in the control arm; the median follow-up time was 365 days [317–365] and 365 days [175–365], corresponding to 4054 person-years and 4515 person-years of follow-up, respectively. The crude IRD between two arms for diabetes rehospitalizations was − 146.02 (− 168.16 to − 123.88) per 1000 person-years. Using nirmatrelvir/ritonavir was associated with a significantly reduced risk of post-COVID diabetes rehospitalizations compared with no treatment (HR = 0.70; 95% CI: 0.60 to 0.81, *p* < 0.001) (Fig. 3).

### Effect of molnupiravir

Analysis on newly onset diabetes included 7601 molnupiravir recipients and 16,117 patients in control arm, with a median follow-up time of 344 days [301–365] and 365 days [239–365], corresponding to 6107 person-years and 12,162 person-years, respectively; analysis on exacerbated diabetes for rehospitalization included 5465 molnupiravir recipients and 7771 patients in control arm, with a median follow-up time of 350 days [304–365] and 365 days [175–365], corresponding to 4391 person-years and 5404 person-years, respectively.

The crude cumulative incidence of newly onset diabetes in the molnupiravir trial was shown in Fig. 2B. There was no significant association between molnupiravir initiation and post-COVID diabetes outcomes (Fig. 3).

### Subgroup, sensitivity, negative control outcome, and post hoc analyses

For both post-COVID diabetes outcomes, the effect sizes of nirmatrelvir/ritonavir and molnupiravir generally remained consistent with those observed in the primary analysis (Additional file 1: Figs. S7 and S8). However, the treatment effect of nirmatrelvir/ritonavir was not statistically significant for female patients, patients aged at or below 65 years, or patients with a CCI of 4 or higher, all of which showed wider confidence intervals. When stratified by vaccination status, the effects reached only borderline statistical significance. A significant additive interaction effect was observed between nirmatrelvir/ritonavir initiation and COVID-19 vaccination against exacerbated diabetes for rehospitalization (RERI: 0.16, 95% CI: 0.01 to 0.30, *p* = 0.033) (Additional file 1: Table S3). The positive RERI indicated that nirmatrelvir/ritonavir conferred a small additional reduction in diabetes rehospitalizations among vaccinated patients with diabetes compared to unvaccinated participants with diabetes.

All sensitivity analyses yielded results that were consistent with those produced in the main analyses (Additional file 1: Fig. S9). The analyses using injury, trauma, or poisoning as outcomes showed treatment strategies had no significant effect on the negative control outcome, suggesting that the observed associations may be less subject to residual confounding (Additional file 1: Fig. S10).

Post hoc analysis showed nirmatrelvir/ritonavir was associated with a significantly lower risk of the composite measure of diabetes progression (diabetes treatment escalation and acute diabetic complications) compared to untreated controls (HR = 0.79; 95% CI: 0.69 to 0.90, *p* < 0.001). In contrast, the effect size was smaller for molnupiravir and of marginal statistical significance (HR = 0.91; 95% CI: 0.81 to 1.03, *p* = 0.147) (Additional file 1: Fig. S11).

## Discussion

Growing evidence implies a possible link between COVID-19 and changes in the pathophysiology of diabetes [44], and studies generally suggest that the diabetes risk is higher in patients with severe COVID-19 during the acute phase, compared to mild infections. Provided that the acute benefits of nirmatrelvir/ritonavir and molnupiravir have been well documented, our target trial emulation study investigated the effectiveness of these antiviral agents on the post-COVID diabetes risk among patients who required hospitalization due to SARS-CoV-2 infection. Primarily, we utilized two analytic cohorts to demonstrate that non-diabetic and diabetic patients with COVID-19 who were prescribed nirmatrelvir/ritonavir had a lower risk of post-acute incidence diabetes and exacerbated diabetes, respectively, compared to those who did not receive any antivirals. Nevertheless, we are unable to demonstrate the effectiveness of molnupiravir on post-COVID diabetes risks in the two different populations. Consistent findings were found across all subgroups in general, although the confidence intervals were widened due to reduced sample sizes. Overall, our results support the long-term benefit of nirmatrelvir/ritonavir and thus suggest prescribing nirmatrelvir/ritonavir over molnupiravir to diabetes patients who were hospitalized due to COVID-19 to reduce the risk of long-term exacerbated diabetes.

Our study demonstrated the effectiveness of nirmatrelvir/ritonavir in reducing the risk of newly onset diabetes among the non-diabetic individuals who required hospitalization for COVID-19. In fact, existing investigations have generally demonstrated a long-term adverse impact of COVID-19 on new diagnoses of diabetes. For example, Rezel-Potts et al. [45] conducted a cohort study among younger adults (median age 35 years) in the UK and reported an increased risk of new-onset diabetes for at least 12 weeks following SARS-CoV-2 infection. Similarly, Naveed et al. [46] analysed a Canadian cohort and demonstrated a greater risk of incident diabetes in the post-acute phase of COVID-19 among a younger population (median age 32 years). Moreover, Xie and Al-Aly [3] used data from the Veterans Health Administration (VHA) in the USA, which included mainly older adults (mean age 60 years), and found an elevated risk and excess burden of post-COVID incident diabetes. According to the findings from the Canadian and VHA cohorts [3, 46], the risk of incidence diabetes is higher among individuals with severe COVID-19 than among those without severe disease. Nevertheless, studies using VHA cohorts (median age 66 years in [17]; mean age 62 years in [18]) have suggested no significant association between nirmatrelvir/ritonavir and incident diabetes among non-hospitalized patients, which contrasts with our findings. This discrepancy may be largely attributed to differences in disease severity across study populations.

Given that the short-term effectiveness of nirmatrelvir/ritonavir against COVID-19 severity has been well documented [7–9], the effect of disease severity triggering the endoplasmic reticulum stress response in β-cells, which leads to apoptosis, may be diminished. The alleviation may lower the risk of a patient transitioning from a prediabetic state to diabetes during the post-acute phase. Apart from that, since severe COVID-19 likely provokes the production of autoantibodies and induces β-cell death [23, 47], the reduced severity brought by nirmatrelvir/ritonavir may help prevent β-cell depletion, which could cause attenuation of pancreatic insulin levels in individuals with insulin-dependent diabetes. These explanations may therefore support our results, which primarily show the benefit of nirmatrelvir/ritonavir in lowering the risk of post-acute incidence diabetes in those without diabetes.

Apart from non-diabetic individuals, we also showed the effectiveness of nirmatrelvir/ritonavir against the long-term exacerbated diabetes in the COVID-19 patients with pre-existing diabetes. While no existing studies have demonstrated the association between nirmatrelvir/ritonavir and post-COVID risk of exacerbated diabetes, several investigations have examined its short-term effectiveness on different outcomes in diabetes patients [19–22]. For example, Lui et al. suggested both molnupiravir users and nirmatrelvir-ritonavir users had a lower risk of 90-day all-cause mortality and hospitalization among patients with type 2 diabetes after SARS-CoV-2 infection, compared to the untreated controls [21]. Similar findings were reported by Wu et al., indicating an association between nirmatrelvir/ritonavir use and a lower risk of all-cause hospitalization or death in non-hospitalized diabetic patients [20]. Our study extends previous findings by demonstrating that nirmatrelvir/ritonavir may prevent diabetes-related hospitalizations (including complications). As mentioned above, we speculated that the effect of nirmatrelvir/ritonavir in reducing acute severity may similarly prevent the long-term metabolic disturbances after an infection, thereby lowering the risk of severe complications related to diabetes that require hospitalization. Further research is needed to fully understand the relationship between nirmatrelvir/ritonavir and the post-COVID risk of exacerbated diabetes.

It should be noted that our study is unable to demonstrate the effectiveness of molnupiravir on post-COVID diabetes risk. Several studies indeed support this finding. In the phase 2–3 MOVe-OUT trial [9], the subgroup analysis showed that molnupiravir recipients had a slightly higher risk of severe COVID-19 in patients with diabetes compared to those receiving placebo, although this result was not statistically significant. In addition, a comparative effectiveness study found that COVID-19 patients with type 2 diabetes who were prescribed nirmatrelvir/ritonavir had a lower risk of 28-day all-cause mortality compared to those prescribed molnupiravir [22]. The observed lack of benefit with molnupiravir, in contrast to the protective effectiveness of nirmatrelvir/ritonavir in the current study, may be attributed to differences in their mechanisms of action and antiviral potency. Nirmatrelvir inhibits the SARS-CoV-2 main protease (Mpro), thereby preventing cleavage of the viral polyprotein and blocking replication [48], whereas molnupiravir induces lethal mutagenesis by being incorporated into the viral RNA during replication, which ultimately impairs viral function [49]. In addition, ritonavir acts as a pharmacokinetic booster for nirmatrelvir, increasing its plasma and tissue concentrations and potentially enhancing its antiviral effect. A previous randomized controlled trial demonstrated that molnupiravir was associated with approximately 25% slower SARS-CoV-2 viral clearance compared with nirmatrelvir/ritonavir in patients with COVID-19 [50]. This superior viral suppression by nirmatrelvir/ritonavir might also limit viral replication in extrapulmonary tissues, including the pancreas, thereby reducing cytopathic damage to pancreatic β-cells caused by SARS-CoV-2 infection and contributing to a lower risk of post-acute COVID-19 diabetes outcomes. Further investigation into the pathophysiological mechanisms underlying these differential long-term effects of the two antivirals is warranted.

One strength of our investigation lies in the use of a large population cohort predominantly infected with the Omicron variant of SARS-CoV-2. The cohort minimizes the impact of variant heterogeneity and reinfections observed in other common cohorts for COVID-19 studies. Since the onset of the pandemic in 2020, Hong Kong has implemented strict public health measures [51, 52], and the majority of SARS-CoV-2 infections only occurred during the Omicron outbreak [53, 54]. Hence, our findings from an Omicron-dominated population are more generalized to newly emerging variants, given a stable trend of mild disease severity and short duration of acute illness across ongoing variants and sub-lineages. Apart from that, we used all hospital admission records from the public sector, which represents about 90% population-wide admissions in Hong Kong. With close clinical monitoring during hospital stays, the validity of the study data, particularly regarding antiviral prescription records, was thoroughly captured.

There are several major limitations in this study. Firstly, despite using the target trial emulation framework on real-world data, the lack of available information on some confounders could lead to residual confounding. For example, some clinical information such as virologic rebound of SARS-CoV-2 [55], lipid parameters, blood glucose measurements, smoking status, and BMI were unavailable at baseline, except for the individuals with regular monitoring of chronic conditions in the public sector. We were also unable to account for potential confounders, including family history of myocardial infarction, diabetes, or autoimmune diseases, and influenza vaccination status, which are typically not available in administrative electronic health record databases. In particular, BMI profiles may have differed systematically between the nirmatrelvir/ritonavir, molnupiravir, and control arms. On one hand, antiviral treatments are recommended and prioritized for patients at high risk of progression to severe COVID-19, including those with obesity. Consequently, patients with higher BMI may have been more likely to receive treatment, which could introduce bias toward the null and potentially underestimate the true beneficial effect of antivirals on reducing the risk of diabetes-related outcomes, given that higher BMI is a major driver of type 2 diabetes. On the other hand, contraindications to nirmatrelvir/ritonavir (e.g., severe renal impairment and severe hepatic impairment) may be more prevalent among patients with higher BMI at baseline, potentially resulting in a lower average BMI in the nirmatrelvir/ritonavir arm than expected from risk prioritization alone. In the absence of BMI data, we cannot determine which effect predominated. Nonetheless, we mitigated this potential residual confounding by indication by including several proxies in the IPCW procedure, including prior use of lipid-lowering drugs (as a surrogate for hyperlipidemia) and comorbidities associated with obesity and smoking (e.g., coronary heart disease, hypertension, chronic pulmonary disease, and cancer). Furthermore, negative control of outcome analyses suggested minimal influence of unmeasured confounders on the observed associations. Secondly, our study did not examine other antivirals, such as remdesivir, due to the relatively low number of recipients. Thirdly, although we have excluded individuals with a known history of diabetes, we cannot dismiss the possibility that some may have been undiagnosed cases that went unrecognized prior to their SARS-CoV-2 infections. Finally, our study included a Hong Kong population of mainly Chinese ethnicity. Therefore, our findings may not be fully generalizable to populations of other ethnicities (e.g., African or Caucasian), where key differences in genetics, socioeconomic factors, and healthcare access could affect post-acute sequelae of COVID-19, such as incident diabetes.

## Conclusions

In conclusion, our study demonstrates the effectiveness of nirmatrelvir/ritonavir in lowering the risk of post-COVID diabetes outcomes among the hospitalized population with COVID-19. Together with existing evidence, our findings suggest that timely treatment with nirmatrelvir/ritonavir not only improves acute COVID-19 severity but also enhances post-COVID diabetes management. Nevertheless, the effectiveness of molnupiravir in mitigating post-COVID diabetes risks could not be demonstrated, and we thus suggest prescribing nirmatrelvir/ritonavir instead of molnupiravir for managing post-COVID diabetes among the hospitalized population, if the patients do not have severe liver or renal impairment.

## Supplementary Information

Additional file 1: Tables S1–S3 and Figures S1–S11. Table S1 Target trial specification and emulation using observational data. Table S2 Code used to define ventilatory support. Table S3 Additive and multiplicative interaction analysis between treatment and age, sex, vaccination status, and CCI. Figure S1 Visualization of diabetes rehospitalizations among patients diagnosed with diabetes before index date. Figure S2 Absolute standardized mean differences of baseline covariates in nirmatrelvir/ritonavir trial for analyzing newly onset diabetes. Figure S3 Absolute standardized mean differences of baseline covariates in molnupiravir trial for analyzing newly onset diabetes. Figure S4 Absolute standardized mean differences of baseline covariates in nirmatrelvir/ritonavir trial for analyzing exacerbated diabetes for rehospitalization. Figure S5 Absolute standardized mean differences of baseline covariates in molnupiravir trial for analyzing exacerbated diabetes for rehospitalization. Figure S6 Schoenfeld residuals plots for main analysis. Figure S7 Subgroup analysis on newly onset diabetes. Figure S8 Subgroup analysis on exacerbated diabetes for rehospitalization. Figure S9 Sensitivity analysis of outcomes. Figure S10 Analysis on negative control of outcomes—injury, trauma, or poisoning. Figure S11 Post hoc analysis on composite measure of diabetes progression.

Additional file 2: TARGET checklist.

## Data Availability

The Hong Kong Hospital Authority and Department of Health, The Government of the Hong Kong Special Administrative Region, are the data custodians, and data requests to these parties can be made via email (hacpaaedr@ha.org.hk) and website (https://www.dh.gov.hk/english/aboutus/aboutus_pps/aboutus_pps.html), respectively. The cases’ surveillance data and medication records were extracted from electronic records in the system managed by the Hong Kong Hospital Authority. The vaccine history was extracted from the COVID-19 surveillance database provided by the Department of Health in Hong Kong. Restrictions apply to the availability of these data, which were used under an agreement for the purposes of scientific research. The authors do not have the right to transfer or release the data, in whole or in part, and in whatever form or media, or to any other parties or place outside of Hong Kong, and must fully comply with the duties under the law relating to the protection of personal data, including those under the Personal Data (Privacy) Ordinance and its principles in all aspects.

## Abbreviations

HR: Hazard ratio
RT-PCR: Reverse transcription polymerase chain reaction
EHR: Electronic health records
HA: Hospital Authority
DH: Department of Health
ICD-9-CM: International Classification of Diseases, Ninth Revision, Clinical Modification
ICPC-2: International Classification of Primary Care, Second Edition
CCI: Charlson Comorbidity Index
ACEI: Angiotensin-converting enzyme inhibitors
IPCW: Inverse probability of censoring weighting
SMD: Standardized mean difference
IRD: Incidence rate difference
CI: Confidence interval
PWP-TT: Prentice, Williams, and Peterson repeated events model for total time
RERI: Relative excess risk for interaction
VHA: Veterans Health Administration
