# Supplementary material for: Effectiveness of nirmatrelvir/ritonavir and molnupiravir on post-COVID diabetes risk among an older adult cohort: a target trial emulation study
Source: BMC Med. 2026 Mar 17;24:227. doi: 10.1186/s12916-026-04791-2 (PMC13069708; doi:10.1186/s12916-026-04791-2)
Supplement: Supplementary file 1 — Additional file 1: Tables S1–S3 and Figures S1–S11. Table S1 Target trial specification and emulation using observational data. Table S2 Code used to define ventilatory support. Table S3 Additive and multiplicative interaction analysis between treatment and age, sex, vaccination status, and CCI. Figure S1 Visualization of diabetes rehospitalizations among patients diagnosed with diabetes before index date. Figure S2 Absolute standardized mean differences of baseline covariates in nirmatrelvir/ritonavir trial for analyzing newly onset diabetes. Figure S3 Absolute standardized mean differences of baseline covariates in molnupiravir trial for analyzing newly onset diabetes. Figure S4 Absolute standardized mean differences of baseline covariates in nirmatrelvir/ritonavir trial for analyzing exacerbated diabetes for rehospitalization. Figure S5 Absolute standardized mean differences of baseline covariates in molnupiravir trial for analyzing exacerbated diabetes for rehospitalization. Figure S6 Schoenfeld residuals plots for main analysis. Figure S7 Subgroup analysis on newly onset diabetes. Figure S8 Subgroup analysis on exacerbated diabetes for rehospitalization. Figure S9 Sensitivity analysis of outcomes. Figure S10 Analysis on negative control of outcomes—injury, trauma, or poisoning. Figure S11 Post hoc analysis on composite measure of diabetes progression. [file 12916_2026_4791_MOESM1_ESM.docx]

**Supplementary materials**

**Table of Contents**

[Table S1. Target trial specification and emulation using observational data. 2](#_Toc219819590)

[Table S2. Code used to define ventilatory support. 7](#_Toc219819591)

[Table S3. Additive and multiplicative interaction analysis between treatment and age, sex, vaccination status, and CCI. 8](#_Toc219819592)

[Figure S1. Visualization of diabetes rehospitalizations among patients diagnosed with diabetes before index date. 10](#_Toc219819593)

[Figure S2. Absolute standardized mean differences of baseline covariates before (red dots) and after weighting (blue dots) between treatment arm and control arm in nirmatrelvir/ritonavir trial for analyzing newly onset diabetes. 11](#_Toc219819594)

[Figure S3. Absolute standardized mean differences of baseline covariates before (red dots) and after weighting (blue dots) between treatment arm and control arm in molnupiravir trial for analyzing newly onset diabetes. 12](#_Toc219819595)

[Figure S4. Absolute standardized mean differences of baseline covariates before (red dots) and after weighting (blue dots) between treatment arm and control arm in nirmatrelvir/ritonavir trial for analyzing exacerbated diabetes for rehospitalization. 13](#_Toc219819596)

[Figure S5. Absolute standardized mean differences of baseline covariates before (red dots) and after weighting (blue dots) between treatment arm and control arm in molnupiravir trial for analyzing exacerbated diabetes for rehospitalization. 14](#_Toc219819597)

[Figure S6. Schoenfeld residuals plots for main analysis. 15](#_Toc219819598)

[Figure S7. Subgroup analysis on newly onset diabetes. 16](#_Toc219819599)

[Figure S8. Subgroup analysis on exacerbated diabetes for rehospitalization. 17](#_Toc219819600)

[Figure S9. Sensitivity analysis of outcomes. 18](#_Toc219819601)

[Figure S10. Analysis on negative control of outcomes - injury, trauma, or poisoning. 20](#_Toc219819602)

[Figure S11. Post-hoc analysis on composite measure of diabetes progression. 21](#_Toc219819603)

Table S1. Target trial specification and emulation using observational data.

| **Protocol component** | **Target trial specification** | **Emulation using observational data** |
| --- | --- | --- |
| **Nirmatrelvir/ritonavir trial** | | |
| Eligibility criteria | Aged over 18 years, with first-time confirmed SARS-CoV-2 infection diagnosis and hospital admission from March 11, 2022 to October 10, 2023.   Exclude patients who:   - Had a history of diabetes before index date (for analysing newly onset diabetes) - Without confirmed diabetes before index date (for analysing exacerbated diabetes for rehospitalization) - Diagnosed with type 1 diabetes (for analysing exacerbated diabetes for rehospitalization) - Died on index date - Received nirmatrelvir/ritonavir or molnupiravir before index date - Had drug contraindications to nirmatrelvir/ritonavir - Had severe renal impairment (i.e., estimated glomerular filtration rate < 30 ml/min per 1·73 m^2^, dialysis, or renal transplantation) - Had severe liver impairment (i.e., cirrhosis, hepatocellular carcinoma, or liver transplantation)   The index date is defined as the date of SARS-CoV-2 diagnosis. | Same as for specification.  The date of confirmed SARS-CoV-2 infection diagnosis is the date of first positive RT-PCR during the enrolment period.  Patients are considered as COVID-19 hospitalizations if they are admitted to hospital within 3 days before or after the date of the confirmation of the SARS-CoV-2 infection. |
| Treatment strategies | Treatment arm: initiation of nirmatrelvir/ritonavir within 5 days of symptom onset according to the FDA-approved regimen. Standard of care is followed in all other respects.  Control arm: standard of care is followed in all other respects.  Patients are expected to complete the full course of drugs.  Physicians can prescribe other concomitant drugs for patients in both treatment and control groups.  Patients in the treatment groups shall be censored if they receive an additional course of molnupiravir after nirmatrelvir/ritonavir treatment. | Treatment arm: initiation of nirmatrelvir/ritonavir within 5 days of symptom onset.  Control arm: without initiation of nirmatrelvir/ritonavir within 5 days of symptom onset and without receipt of molnupiravir.  Date of positive RT-PCR is used as a proxy of date of symptom onset.  It is assumed that once the patient initiated antiviral therapy, he/she will complete the full course of antiviral treatment.  Concomitant treatments at baseline are adjusted in the model. |
| Assignment procedures | Patients are assigned randomly to one of the treatment strategies and are informed of the assigned strategy. | Patients are classified into different treatment groups according to the prescription records within 5 days of index date.  Randomization of treatment assignments is emulated by cloning, censoring, and inverse probability of censoring weighting to address immortal time bias and post-assignment confounders. |
| Follow-up | For each person, follow-up starts on the day of treatment randomization and ends at the occurrence of outcome events (only for the analysis of newly onset diabetes), death, 1 year after the index date, or the end of data availability (October 31, 2023), whichever comes first. | Same as for specification. |
| Outcomes | Incident diabetes occurred from 21-365 days following SARS-CoV-2 infection. Exacerbated diabetes for rehospitalizations. | Same as for specification. |
| Causal contrast | Per-protocol effect.  Intention-to-treat effect. | Observational analogue of per-protocol effect. |
| Identifying assumptions | Within levels of the adjustment of baseline confounders, groups receiving each treatment strategy at each time have the same counterfactual risk of the outcome (conditional exchangeability) and there is a non-zero probability of receiving each treatment strategy at each time.  For every combination of the baseline covariates, there is a non-zero probability of receiving each treatment strategy at each time.  The treatment strategies are sufficiently well defined. | Same as for specification.  Cloning, censoring and inverse probability of censoring weighting are used to address informative censoring and baseline confounders.  Negative control of outcomes approach is applied to detect residual confounding effect caused by unobserved confounders. |
| Data analysis | Measure the cause-specific hazard ratio of incident diabetes and recurrent diabetes rehospitalizations across treatment strategies. | Estimate the cause-specific hazard ratio of cardiovascular complications with inverse probability of censoring weighting considered and death as a competing risk by using the cause-specific Cox proportional hazard model. |
| **Molnupiravir trial** | | |
| Eligibility criteria | Aged over 18 years, with first-time confirmed SARS-CoV-2 infection diagnosis and hospital admission from March 11, 2022 to October 10, 2023.   Exclude patients who:   - Had a history of diabetes before index date (for analysing newly onset diabetes) - Without confirmed diabetes before index date (for analysing exacerbated diabetes for rehospitalization) - Diagnosed with type 1 diabetes (for analysing exacerbated diabetes for rehospitalization) - Dead on index date - Received nirmatrelvir/ritonavir or molnupiravir before index date - Had drug contraindications to nirmatrelvir/ritonavir   The index date is defined as the date of SARS-CoV-2 diagnosis. | Same as for specification.  The date of confirmed SARS-CoV-2 infection diagnosis is the date of first positive RT-PCR during the enrolment period.  Patients are considered as COVID-19 hospitalizations if they are admitted to hospital within 3 days before or after the date of the confirmation of the SARS-CoV-2 infection. |
| Treatment strategies | Treatment arm: initiation of molnupiravir within 5 days of symptom onset according to the FDA-approved regimen. Standard of care is followed in all other respects.  Control arm: standard of care is followed in all other respects  Patients are expected to complete the full course of drugs.  Physicians can prescribe other concomitant drugs for patients in both treatment and control groups.  Patients in the treatment groups shall be censored if they receive an additional course of nirmatrelvir/ritonavir after molnupiravir treatment. | Treatment arm: initiation of molnupiravir within 5 days of symptom onset.  Control arm: without initiation of molnupiravir within 5 days of symptom onset and without receipt of nirmatrelvir/ritonavir.  Date of positive RT-PCR is used as a proxy of date of symptom onset.  It is assumed that once the patient initiated antiviral therapy, he/she will complete the full course of antiviral treatment.  Concomitant treatments at baseline are adjusted in the model. |
| Assignment procedures | Patients are assigned randomly to one of the treatment strategies and are informed of the assigned strategy. | Patients are classified into different treatment groups according to the prescription records within 5 days of index date.  Randomization of treatment assignments is emulated by cloning, censoring, and inverse probability of censoring weighting to address immortal time bias and post-assignment confounders. |
| Follow-up | For each person, follow-up starts on the day of treatment randomization and ends at the occurrence of outcome events (only for the analysis of newly onset diabetes), death, 1 year after the index date, or the end of data availability (October 31, 2023), whichever comes first. | Same as for specification. |
| Outcomes | Incident diabetes occurred from 21-365 days following SARS-CoV-2 infection. Exacerbated diabetes for rehospitalizations. | Same as for specification. |
| Causal contrast | Per-protocol effect.  Intention-to-treat effect. | Observational analogue of per-protocol effect. |
| Identifying assumptions | Within levels of the adjustment of baseline confounders, groups receiving each treatment strategy at each time have the same counterfactual risk of the outcome (conditional exchangeability) and there is a non-zero probability of receiving each treatment strategy at each time.  For every combination of the baseline covariates, there is a non-zero probability of receiving each treatment strategy at each time.  The treatment strategies are sufficiently well defined. | Same as for specification.  Cloning, censoring and inverse probability of censoring weighting are used to address informative censoring and baseline confounders.  Negative control of outcome approach is applied to detect residual confounding effect caused by unobserved confounders. |
| Data analysis | Measure the cause-specific hazard ratio of incident diabetes and recurrent diabetes rehospitalizations across treatment strategies. | Estimate the cause-specific hazard ratio of post-COVID diabetes with inverse probability of censoring weighting considered and death as a competing risk by using the cause-specific Cox proportional hazard model. |

Table S2. Code used to define ventilatory support.

| **Procedure** | **Code (ICD-9)** |
| --- | --- |
| Extracorporeal membrane oxygenation (ECMO) | 39.65 |
| Other sleep disorder function tests | 89.18 |
| Non-invasive mechanical ventilation | 93.9 |
| Hyperbaric oxygenation | 93.95 |
| Other oxygen enrichment | 93.96 |
| Other continuous invasive mechanical ventilation | 96.7x |
| Insertion of endotracheal tube | 96.04 |

Table S3. Additive and multiplicative interaction analysis between treatment and age, sex, vaccination status, and CCI.

| ***Age*** | | | | | | |
| --- | --- | --- | --- | --- | --- | --- |
| **Outcome** | **RERI estimate** | **95% CI** | **P value** | **Multiplicative estimate** | **95% CI** | **P value** |
| Newly onset diabetes |  |  |  |  |  |  |
| Nirmatrelvir/ritonavir trial | -0.04 | (-1.08 to 0.99) | 0.935 | 1.02 | (0.70 to 1.49) | 0.915 |
| Molnupiravir trial | -0.16 | (-0.82 to 0.51) | 0.644 | 0.84 | (0.64 to 1.09) | 0.182 |
| Exacerbated diabetes for rehospitalization | | |  |  |  |  |
| Nirmatrelvir/ritonavir trial | -0.09 | (-0.28 to 0.11) | 0.380 | 0.92 | (0.72 to 1.17) | 0.492 |
| Molnupiravir trial | -0.15 | (-0.35 to 0.05) | 0.134 | 0.85 | (0.68 to 1.07) | 0.165 |
| ***Sex*** | | | | | | |
| **Outcome** | **RERI estimate** | **95% CI** | **P value** | **Multiplicative estimate** | **95% CI** | **P value** |
| Newly onset diabetes |  |  |  |  |  |  |
| Nirmatrelvir/ritonavir trial | 0.01 | (-0.56 to 0.57) | 0.981 | 1.03 | (0.71 to 1.50) | 0.869 |
| Molnupiravir trial | -0.26 | (-0.59 to 0.08) | 0.140 | 0.81 | (0.62 to 1.05) | 0.107 |
| Exacerbated diabetes for rehospitalization | | |  |  |  |  |
| Nirmatrelvir/ritonavir trial | -0.08 | (-0.26 to 0.09) | 0.369 | 0.91 | (0.72 to 1.16) | 0.468 |
| Molnupiravir trial | -0.16 | (-0.36 to 0.04) | 0.111 | 0.85 | (0.68 to 1.06) | 0.145 |
| ***Vaccination*** | | | | | | |
| **Outcome** | **RERI estimate** | **95% CI** | **P value** | **Multiplicative estimate** | **95% CI** | **P value** |
| Newly onset diabetes |  |  |  |  |  |  |
| Nirmatrelvir/ritonavir trial | 0.11 | (-0.13 to 0.35) | 0.367 | 1.07 | (0.73 to 1.57) | 0.725 |
| Molnupiravir trial | -0.12 | (-0.33 to 0.08) | 0.240 | 0.85 | (0.66 to 1.11) | 0.240 |
| Exacerbated diabetes for rehospitalization | | |  |  |  |  |
| Nirmatrelvir/ritonavir trial | 0.16 | (0.01 to 0.30) | 0.033 | 0.95 | (0.74 to 1.21) | 0.658 |
| Molnupiravir trial | -0.10 | (-0.27 to 0.07) | 0.235 | 0.85 | (0.68 to 1.07) | 0.172 |
| ***CCI*** | | | | | | |
| **Outcome** | **RERI estimate** | **95% CI** | **P value** | **Multiplicative estimate** | **95% CI** | **P value** |
| Newly onset diabetes |  |  |  |  |  |  |
| Nirmatrelvir/ritonavir trial | -0.43 | (-1.31 to 0.44) | 0.331 | 1.03 | (0.71 to 1.48) | 0.884 |
| Molnupiravir trial | -0.27 | (-0.98 to 0.44) | 0.451 | 0.84 | (0.65 to 1.09) | 0.182 |
| Exacerbated diabetes for rehospitalization | | |  |  |  |  |
| Nirmatrelvir/ritonavir trial | -0.21 | (-0.52 to 0.10) | 0.180 | 0.93 | (0.73 to 1.18) | 0.536 |
| Molnupiravir trial | -0.27 | (-0.65 to 0.11) | 0.163 | 0.86 | (0.68 to 1.07) | 0.176 |

RERI: relative excess risk for interaction. CI: confidence interval. CCI: Charlson Comorbidity Index. For interaction analyses, binary coding was applied: patients aged ≤65 years, female patients, unvaccinated patients, or those with CCI ≤4 were assigned 0, with their respective counterparts assigned 1. Treatment assignment was coded as 1 (treatment arm) or 0 (control arm) based on status at the end of the grace period. Cox models included the product term of these variables. Additive interaction was assessed using RERI, while multiplicative interaction was quantified by exponentiating the product term's coefficient. A two-sided Wald test was used for statistical inference.

****Figure S1. Visualization of diabetes rehospitalizations among patients diagnosed with diabetes before index date. **(A) Rehospitalizations plot for patients in nirmatrelvir/ritonavir trial. (B) Rehospitalizations plot for patients in molnupiravir trial.** The grey horizontal bars represented each patients’ follow-up time. Bars were stacked and sorted based on the ascending order of follow-up time. Green solid points represented the rehospitalizations due to diabetes.

****Figure S2. Absolute standardized mean differences of baseline covariates before (red dots) and after weighting (blue dots) between treatment arm and control arm in nirmatrelvir/ritonavir trial for analyzing newly onset diabetes. The solid line marks a SMD of 0 and the dash line marks a SMD of 0.1.

****Figure S3. Absolute standardized mean differences of baseline covariates before (red dots) and after weighting (blue dots) between treatment arm and control arm in molnupiravir trial for analyzing newly onset diabetes. The solid line marks a SMD of 0 and the dash line marks a SMD of 0.1.

****Figure S4. Absolute standardized mean differences of baseline covariates before (red dots) and after weighting (blue dots) between treatment arm and control arm in nirmatrelvir/ritonavir trial for analyzing exacerbated diabetes for rehospitalization. The solid line marks a SMD of 0 and the dash line marks a SMD of 0.1.

****Figure S5. Absolute standardized mean differences of baseline covariates before (red dots) and after weighting (blue dots) between treatment arm and control arm in molnupiravir trial for analyzing exacerbated diabetes for rehospitalization. The solid line marks a SMD of 0 and the dash line marks a SMD of 0.1.

****Figure S6. Schoenfeld residuals plots for main analysis. Circles represented Schoenfeld residuals and the solid line represented smoothing spline fit to the plot. P values were computed from the Schoenfeld residuals test. The dashed lines represented a +/- 2-standard-error band around the fit.

****Figure S7. Subgroup analysis on newly onset diabetes. (A) nirmatrelvir/ritonavir. (B) Molnupiravir trial. Crude incidence rate difference was calculated for treatment arm and control arm before inverse probability of censoring weighting. Adjusted hazard ratios were estimated using weighted cause-specific Cox proportional hazard models.

IRD: incidence rate difference. HR: hazard ratio. CI: confidence interval.

****Figure S8. Subgroup analysis on exacerbated diabetes for rehospitalization. (A) nirmatrelvir/ritonavir. (B) Molnupiravir trial. Crude incidence rate difference was calculated for treatment arm and control arm before inverse probability of censoring weighting. Adjusted hazard ratios were estimated using weighted cause-specific Cox proportional hazard models.

IRD: incidence rate difference. HR: hazard ratio. CI: confidence interval.

****Figure S9. Sensitivity analysis of outcomes. Crude incidence rate difference was calculated for treatment arm and control arm before inverse probability of censoring weighting. Adjusted hazard ratios were estimated using weighted cause-specific Cox proportional hazard models.

IRD: incidence rate difference. HR: hazard ratio. CI: confidence interval. PWP-TT: Prentice, Williams, and Peterson repeated events model for total time.

****Figure S10. Analysis on negative control of outcomes - injury, trauma, or poisoning. Crude incidence rate difference was calculated for treatment arm and control arm before inverse probability of censoring weighting. Adjusted hazard ratios were estimated using weighted cause-specific Cox proportional hazard models.

IRD: incidence rate difference. HR: hazard ratio. CI: confidence interval.

Figure S11. Post-hoc analysis on composite measure of diabetes progression. Crude incidence rate difference was calculated for treatment arm and control arm before inverse probability of censoring weighting. Adjusted hazard ratios were estimated using weighted cause-specific Cox proportional hazard models.

IRD: incidence rate difference. HR: hazard ratio. CI: confidence interval.
